# Associations between nursing home leadership turnover and resident satisfaction

**DOI:** 10.1093/geront/gnag106

**Published:** 2026-05-11

**Authors:** Jenny H Kwon, John R Bowblis

**Affiliations:** Philip R. Lee Institute for Health Policy Studies, University of California, San Francisco, California, United States; Department of Epidemiology & Biostatistics, University of California, San Francisco, California, United States; Department of Economics, Farmer School of Business, Miami University, Oxford, Ohio, United States; Scripps Gerontology Center, Miami University, Oxford, Ohio, United States

**Keywords:** Workforce issues, Quality of life, Long-term care, Caregiving—formal

## Abstract

**Background and Objectives:**

The impact of nursing home administrator (NHA) and director of nursing (DON) turnover on residents’ care experiences remains underexplored. This study aims to examine the associations of NHA/DON turnover with resident satisfaction scores.

**Research Design and Methods:**

5 data sources were merged to create an aggregate dataset of 752 Ohio nursing homes in 2017: the Ohio Nursing Home Resident Satisfaction Survey, the Ohio Biennial Survey of Long-Term Care Facilities, the Certification and Survey Provider Enhanced Reports, Medicaid Cost Report, and LTCFocus. Resident satisfaction was measured by 8 resident satisfaction scores: the overall satisfaction score and 7 individual domain scores. The scores were continuous variables ranging from 0 (low) to 100 (high). Leadership turnover was assessed using 4 specifications: 1 NHA turnover, 2 or more NHA turnovers, 1 DON turnover, and 2 or more DON turnovers over the past 3 years. Multiple linear regressions were conducted via SAS, adjusting for control variables.

**Results:**

2 or more DON turnovers are associated with a lower overall resident satisfaction score by 1.14% points (*p* < .05), lower Environment domain score by 1.01% points (*p* < .05), and lower Caregivers domain score by 1.80% points (*p* < .01). NHA turnover is not statistically associated with overall resident satisfaction score but positively associated with 2 individual domain scores.

**Discussion and Implications:**

DON turnover is associated with lower resident satisfaction scores, while NHA turnover is not. A single DON turnover may not harm residents’ care experiences; however, frequent turnover among DON could negatively impact them.

## Background

Nursing homes (NHs) in the U.S. are criticized for not providing high-quality care and a good quality of life for residents (Allen et al., 2006; Hansen et al., 2019). Despite efforts by state and federal agencies to improve NH quality, a major critique of the current quality reporting system is that consumers’ voices about NH quality are missing (Shippee et al., 2023; Williams et al., 2016). For example, the Five-Star Quality Rating System, a nationwide NH quality report provided by the Centers for Medicare & Medicaid Services (CMS), does not capture the satisfaction of residents or families with their experiences in the NH when calculating ratings (Centers for Medicare & Medicaid Services [CMS], 2018). The overall star rating is calculated based on the NH health inspection, residents’ function and health status, and staffing levels (CMS, 2018), but does not incorporate consumers’ perspectives (Williams et al., 2016).

Similarly, most previous studies on NH quality have focused on clinical outcomes such as falls and mortality without the benefits of consumer input (Raymond et al., 2025; Sanghavi et al., 2020). While some items in the federally mandated NH Minimum Data Set 3.0 (e.g., Preference Assessment Tool in Section F) and deficiency citations related to residents’ rights measure non-clinical aspects of residents’ living in NHs, a recent study found that those measures are not closely associated with the resident-rated quality of life scores (Shippee et al., 2023). This suggests that the existing federally collected measures of quality of life cannot substitute for consumer-rated quality measures. Recently, there have been increasing calls to collect NH consumer-derived quality data (National Academies of Sciences, Engineering, and Medicine [NASEM], 2022) to fill this gap in quality measurement. In response, this study investigates the association between NH leadership turnover and resident satisfaction, a key consumer-derived measure of quality.

### Quality of life in NHs

NH residents’ quality of life is an overarching concept that represents an array of aspects of NH living (Kane, 2001). It encompasses residents’ physical and cognitive functioning, psychological well-being, autonomy, life satisfaction, social participation, self-esteem, level of energy or fatigue, distress, emotions, and feelings (Franklin et al., 2006; Kane, 2001; Shippee, 2012). Although the importance of NH residents’ quality of life has gained much attention in recent decades, research in this area has been limited due to challenges in measuring the concept (Shippee et al., 2023). The absence of reliable measures and a consolidated reporting system has limited the availability of consistent data for both research and quality improvement initiatives.

### NH resident satisfaction

NH resident satisfaction is a subjective assessment of how content or pleased residents are with specific services, care delivery, and environment provided (Applebaum et al., 1999). In contrast, quality of life is a broader concept that assesses various aspects of residents’ physical, emotional, social, and environmental well-being (Kane, 2001). While quality of life reflects a holistic view of residents’ overall well-being, resident satisfaction specifically zeros in on their experiences with service-related aspects and perceptions of NH care (Straker et al., 2007). NH resident satisfaction is typically assessed through surveys that ask residents about their experiences with aspects such as staff responsiveness, cleanliness, food quality, social activities, and overall care (Straker et al., 2007).

NH resident satisfaction is shaped by multiple factors within their living environment, each playing a critical role in influencing their overall well-being (Shippee et al., 2015; Wheatley et al., 2007). These factors include the welcome and informed moving-in experience, the safety and cleanliness of the building, the availability of meal choices, opportunities for activities and recreational time, the responsiveness and quality of staff interactions, and the overarching culture within NHs (Zinn et al., 1993). While some of these elements have been studied extensively, others remain underexplored. Nevertheless, these different factors simultaneously interact and contribute to the complex dynamics that shape resident experiences (Chou et al., 2002).

While resident satisfaction and quality of life are related, they are distinct concepts that differ in scope and measurement. A high satisfaction score does not always indicate a high quality of life. For example, a resident may report being satisfied with specific services but still experience emotional distress or loneliness. In addition, residents may report high satisfaction due to low expectations of services, even if their overall quality of life is poor.

However, resident satisfaction plays a critical role in shaping the quality of life. Unlike one-time service consumers, NH residents rely on care and support continuously throughout their daily lives. Since NH residents are surrounded by the NH environment, their experiences with care services have a profound impact on their overall well-being (Applebaum et al., 1999). Moreover, due to the lack of widely used, tangible tools to measure quality of life among NH residents, the two concepts are often used interchangeably (Tellis-Nayak et al., 2010; Wheatley et al., 2007).

Currently, only two states, Ohio and Minnesota, regularly collect resident-rated data using validated tools (Shippee et al., 2015; Wheatley et al., 2007) due to the absence of federally-administered quality of life or other comparable measures. The Ohio Nursing Home Resident Satisfaction Survey was developed by the Scripps Gerontology Center at Miami University, and the survey is required for all NHs that accept Medicare or Medicaid payments in Ohio (Vital Research, 2018). The survey includes the overall score and seven individual domain scores (i.e., Environment, Moving In, Care and Services, Facility Culture, Caregivers, Spending Time, and Meals and Dining). The Minnesota Resident Quality of Life Survey was created by scholars at the University of Minnesota and has been validated and utilized in Minnesota since 2005 (Shippee et al., 2015). The survey includes an overall quality score and six individual domain scores (i.e., Facility Environment, Attention, Food Enjoyment, Engagement, Negative Mood, and Positive Mood).

A comparison of Ohio’s resident satisfaction survey with Minnesota’s quality of life survey demonstrates that consumer satisfaction surveys of this kind can serve as valid proxies for the broader construct of quality of life. Although the two surveys have different names, they share similar items (Shippee et al., 2015; Straker et al., 2019b). For example, the Ohio survey item “Is it easy for you to get around in your room?” under the Environment domain aligns with the Minnesota survey item “Is it easy for you to get around in your room by yourself?” Other items, such as “Do you like the food here?” and “Do you get your favorite foods here?” are identical in wording across both surveys. In addition, the Ohio survey goes beyond assessing NH service to cover quality of life aspects. For example, the items “Do the people who work here keep you connected to the community?” and “Do you feel included in life here?” reflect residents’ social well-being. Due to these similarities, the Ohio survey has been used as a proxy for quality of life in previous studies (Shippee et al., 2022, 2023). Therefore, this study focuses on resident satisfaction as a key driver of residents’ quality of life.

### NH leadership turnover

Turnover is one of the measures of the NH workforce stability. While the retention rate focuses on how long the existing workforce stays in the same NH, turnover measures how many workers leave the NH within a given period (Kovner et al., 2014). Turnover refers to the proportion of (existing and newly employed) staff who leave the NH in a specified time period (Castle, 2006). As such, there is no upper limit for turnover rate, and it can exceed 100%.

NHs have two primary leadership roles. The first is the nursing home administrator (NHA), who oversees the administrative and operational functions of the NH. Typical duties include supervision of human resources, managing financial resources, addressing consumer feedback, and ensuring resident rights (NASEM, 2022). An earlier study found that the average tenure of NHA was 3.5 years, and annual NHA turnover rates exceeded 40% (Castle, 2001). NHAs with lower job satisfaction were more likely to leave within a year (Castle et al., 2007).

The second key leadership position is the director of nursing (DON). The DON must be a registered nurse, and they serve as the chief nursing manager with overarching responsibility for clinical care (Pradhan et al., 2024b; Rao & Evans, 2015). The DON’s duties can encompass a broad range of nursing management functions, including staff hiring, supervision, and education; budgeting and cost management; ensuring regulatory compliance; and leading quality improvement efforts (Sherman & Touhy, 2017; Siegel et al., 2015). Nationwide, the tenure of DONs is 6.8 years on average, and 42% leave the position within a year (Amirkhanyan et al., 2020).

High turnover of NH leaders has a consistently negative effect on NH quality. Higher NHA turnover is associated with a decreased operating margin (Pradhan et al., 2024a), a low retention rate of certified nursing assistants (CNAs) (Castle, 2005), a higher number of deficiencies (Castle, 2001), and lower star ratings (Castle & Decker, 2011; Zheng et al., 2022). Similarly, lower DON turnover is associated with higher quality-measure star ratings (Krause, 2012). NHs with a DON who reports a higher intention to quit have more deficiencies (McKinney et al., 2016). However, few studies have examined the impact of leadership turnover on resident satisfaction.

### Current study

The purpose of the study is to examine the association between NH leadership turnover and resident satisfaction. It is hypothesized that NHs with higher NHA/DON turnover will receive lower overall resident satisfaction scores as well as lower scores across seven individual domains (H1). Second, given that DONs directly supervise nursing staff and oversee day-to-day care delivery, their turnover is expected to have a greater impact on resident satisfaction than that of NHAs, whose responsibilities are primarily administrative (H2). Furthermore, while both NHA and DON turnover are hypothesized to be negatively associated with resident satisfaction, their effects are expected to vary across domains. Specifically, their turnover is expected to show a stronger association with the Caregivers domain than with domains related to food or activities (H3).

This study contributes to existing literature in several ways. First, it advances understanding of NH quality from the consumers’ perspectives. Previous studies have relied on provider-reported quality data because of the scarcity of consumer-derived NH quality measures (Shippee et al., 2017). The current study fills this knowledge gap by using the resident satisfaction scores collected using validated measures. Moreover, this study investigates both overall resident satisfaction score and individual domain scores, addressing a limitation of prior research that has typically focused only on overall satisfaction (Kwon & Bowblis, 2024; Kwon et al., 2024; Williams et al., 2016). This study also broadens our understanding of NH quality by examining the nuanced effects of leadership turnover on resident satisfaction. Prior research has largely operationalized NHA and DON turnover as a binary measure, capturing only whether turnover occurred within a given period (Kwon & Bowblis, 2024; Kwon et al., 2024). The present study extends this literature by examining the number of leadership changes, thereby assessing whether the frequency or persistence of turnover shapes NH quality outcomes. Given that NHA and DON roles differ in their proximity to and influence on resident care, their turnover may have distinct impacts on residents’ care experiences.

### Conceptual framework

This study is grounded in the Person-Environment (P-E) Fit theory, particularly the Competence-Press Model (Lawton, 1982). P-E Fit posits that an individual’s behavior is determined by the interaction between a person and the environment. Particularly, one’s behavior is a function of the person’s level of competence (i.e., physical, cognitive, emotional, and social capabilities) and environmental press (i.e., challenges or demands presented by the surrounding environment) (Lawton, 1982). The environment encompasses all external factors, including physical, social, and cultural contexts.

An optimal status, such as maximum comfort or positive emotions, is achieved when individuals maintain a similar level of personal competence and environmental press (Lawton, 1982). It is, thus, considered ideal to find a balance between competence and environmental press where people feel most comfortable and competent (Lawton, 1982). For example, older adults who achieve a match between their physical capacity and external challenges feel positive emotions; otherwise, they would feel bored or overchallenged.

In the context of NHs, residents typically have lower and often declining levels of physical and cognitive functioning, which makes them particularly sensitive to changes in their environment. To provide higher satisfaction to residents, it is essential to adjust the environment to align with residents’ shifting competencies. Leadership positions, including the NHA and DON, constitute a key component of the environmental press experienced by residents. Stable leadership ensures consistent oversight and coordination of care, while providing ongoing support for culture change initiatives (Castle, 2001; Corazzini et al., 2015), thereby fostering a supportive environment that aligns with residents’ competence levels.

## Research design and methods

### Data source

To conduct a cross-sectional analysis using secondary data, an aggregate dataset of Ohio NHs in 2017 was constructed by merging five data sources.

The first dataset was the 2017 Ohio Nursing Home Resident Satisfaction Survey, which was required to be completed by all NHs per state law (Straker et al., 2019b). The survey assesses NH residents’ experiences across seven domains of daily living. An example item for each domain is as follows. Environment: Do you feel you have enough privacy? Moving In: Were you given enough help to learn how things work here?; Care and Services: Are your preferences about daily routines carried out?; Facility Culture: Are you encouraged to speak up about things you don’t like here?; Caregivers: Do the people who work here do things the way you want them done?; Spending Time: Do you usually enjoy how you spend your time? Meals and Dining: Do you look forward to mealtimes? All survey items are shown in Supplementary Table 1. The survey has high reliability, having Cronbach’s alpha of 0.78.

The resident survey was conducted via face-to-face structured interviews with residents between August and December 2017. Under a contractual relationship with the Ohio Department of Aging, Vital Research Inc. collected the survey data. A total of 23,154 residents completed it, and their responses were aggregated into NH-level data to construct resident satisfaction scores. On average, 24 surveys were collected from each NH, ranging from 5 to 35 surveys depending on the NH size.

The second dataset, the 2017 Ohio Biennial Survey of Long-Term Care Facilities (The Biennial Survey), was the source of NHA/DON turnover and several control variables. The Biennial Survey was developed and administered by the Scripps Gerontology Center at Miami University, with financial support from the Ohio Department of Aging. All Ohio NHs were required to conduct the Biennial Survey per state law. The purpose of the survey was to collect comprehensive NH-level information in Ohio. The survey consists of several modules covering different items ranging from basic information about NHs and their residents to administrative issues in the industry. The survey was completed by the NH administrator via a self-administered online survey. The survey was collected in 2018, but administrators were asked to report information reflecting the 2017 calendar year. The response rate for the 2017 survey was 91% (Applebaum et al., 2020).

Additional control variables were obtained from three different data sources. For example, NH structural characteristics and resident acuity were from the Certification and Survey Provider Enhanced Reports. Financial resource characteristics and staffing levels were drawn from the Medicaid Cost Report. Resident demographic information was obtained from LTCFocus. Detailed data sources and a summary of measures are presented in Supplementary Table 2.

### Sample

Of the 968 NHs operating in Ohio in 2017, 11 NHs affiliated with hospitals were excluded since those NHs serve mainly short-term patients (Jester et al., 2020). Among 957 non-hospital-based NHs, observations that had any missing values on the key variables were dropped. Thus, the final analytic sample included 752 NHs, representing 78% of NHs operating in 2017.

To avoid sampling bias, the independent sample *t*-tests and chi-square tests were conducted comparing characteristics between NHs that are included in the analytic sample and those excluded. The results found statistically significant differences in some variables between the two groups. However, it is important to note that the substantive significance (i.e., the effect size) was relatively modest. For example, NHs in the analytic sample are less likely to operate as for-profit (79% vs 86%), have fewer resident days covered by Medicare (10% vs 13%), and more CNA staffing levels (2.2 hr vs 2.0 hr) compared to NHs excluded from the analytic sample.

While it is important to use the most current information to understand the NH industry today, key datasets used in this study, such as the Resident Satisfaction Survey, were not collected during the pandemic. Thus, 2017 reflects the most recent data in which resident satisfaction is available.

### Measures

#### Resident satisfaction scores

Eight resident satisfaction scores (overall satisfaction score + seven individual domain scores) were used as dependent variables. The survey has 44 items in total: seven items for the Environment domain, three items for the Moving In domain, six items for the Care and Services domain, eight items for the Facility Culture domain, eight items for the Caregivers domain, seven items for the Spending Time domain, and five items for the Meals and Dining domain (Supplementary Table 1). As the survey was administered as a structured interview, there were three response options that residents could choose: 1 = Generally yes, 2 = Generally no, and 3 = Don’t know/Not applicable. To convert scores into a 100-point scale, each response was re-coded as follows: 1 = 100, 2 = 0, and 3 = missing (Vital Research, 2018).

Seven individual domain scores were calculated as the mean score of items under a certain domain, and the overall satisfaction score was calculated as the mean score of the total 44 items. In this study, the eight scores were used as continuous variables, ranging from 0 to 100. A higher score indicates greater resident satisfaction.

#### NHA/DON turnover

NHA/DON turnover is a key predictor of this study. They are measured by asking how many individuals held each position between 2015 and 2017. To examine whether the number of leadership changes matters, four turnover specifications were created: (a) one NHA turnover, (b) two or more NHA turnovers, (c) one DON turnover, and (d) two or more DON turnovers between 2015 and 2017. This categorization divides NHs into three approximately equal-sized groups, around 30% of NHs in each (Table 2).

#### Control variables

Four sets of explanatory variables that could influence resident satisfaction were controlled for the regression analyses. Specification of measures is in Supplementary Table 2.

First, NH structural characteristics include the number of certified beds, percentage of private rooms, ownership status, chain affiliation, continuing care retirement community (CCRC) affiliation, presence of a memory care unit/special care units, and geographic location. Previous studies consistently reported that NHs with higher certified beds, a lower percentage of private rooms, and for-profit or chain-affiliated are associated with poorer quality (Kennedy, 2023; Lucas et al., 2007; Shippee et al., 2015). The presence of a memory care unit or special care units could enhance NH resident satisfaction by addressing specific care needs and improving personalized care delivery (Straker et al., 2019a). In this study, the not-for-profit NHs and government-owned NHs were collapsed into one group because the number of government-owned NHs is too small to run statistical analysis.

Second, financial resource characteristics, including occupancy rate, Medicare, and Medicaid payer-mix, also impact NH quality. These factors reflect the resource availability to meet residents’ diverse care needs (Lucas et al., 2007). As Medicare reimburses at a higher rate than Medicaid (Atkins et al., 2017), NHs with a Medicare-heavy payer mix often provide better quality than those with a Medicaid-heavy payer mix (Gandhi et al., 2021; Kunkel et al., 2024).

Another factor is staff characteristics, such as staffing levels measured by hours per resident day (HPRD) for nursing staff (e.g., licensed nurses and CNAs) and other staff, such as food service staff, activity staff, and social workers. The use of agency nursing staff and the annual retention rate of nursing staff are also included. Since NHs are a highly labor-intensive industry and staff interact closely with residents every day, higher staffing levels are generally associated with better quality (Bowblis et al., 2023). The use of agency nursing staff could be associated with poorer quality outcomes (Bowblis et al., 2024).

Lastly, resident characteristics such as resident demographic information and case-mix are controlled, as prior research has consistently shown that resident characteristics significantly influence NH quality outcomes. Resident demographic information includes age, gender, and race/ethnicity. Previous research has demonstrated that NHs with a higher proportion of older residents received higher resident satisfaction (Kennedy, 2023) and fewer complaints (Kunkel et al., 2024). Research has found that racial and ethnic minority residents tend to report lower quality of life than their White counterparts (Bowblis et al., 2021; Shippee et al., 2020).

Resident case-mix includes acuity index, the percentage of residents with depression, dementia, psychiatric illness, and intellectual disability. Acuity index is an indexed score indicating residents’ physical ability to perform activities of daily living and the need for special treatment. A higher score indicates a more severe condition and a greater need for specialized care (Cowles, 2015). Residents with limited physical and cognitive abilities reported lower quality of life (Abrahamson et al., 2012; Lucas et al., 2007).

### Analysis

First, summary statistics were assessed for all variables included in the analysis. Out-of-range values were excluded, and the distribution of the eight resident satisfaction scores was assessed to confirm normality and to meet the assumptions of the linear regression. Prior to conducting statistical tests, an advanced diagnostic was performed. Using residual plots, linear regression assumptions (i.e., linearity, homoscedasticity, and independence) were confirmed. Influential observations or outliers were identified using three complementary diagnostics: Cook’s distance, which measures the overall influence of each observation on the regression coefficients; Difference in Betas (DFBETAs), which assesses how much each coefficient changes when a given observation is removed; and Difference in Fits (DFFITS), which captures the degree to which each observation affects its own fitted value. Multicollinearity was checked using variance inflation factors (≥2.5), confirming that none of the independent variables have a high correlation.

A total of eight multiple linear regression models were fitted, including all four NHA and DON turnover predictors and control variables. As each of the resident satisfaction domains is likely to be influenced by different sets of control variables, eight regression models were fitted using slightly different combinations of control variables. Specifically, food service staff HPRD was adjusted for the overall score and the Caregivers and Meals and Dining domains. Activity staff HPRD was adjusted for the overall score, as well as the Care and Services, Caregivers, and Spending Time domains. The remaining control variables were consistently adjusted across the eight resident satisfaction scores. A two-sided *p-*value of <.05 was used as the criterion for statistical significance. All analyses were conducted via SAS version 9.4.

## Results

### Summary statistics

Table 1 shows the summary statistics of eight resident satisfaction scores. The mean overall score was 76.5 (*SD *= 5.9) with a range from 54.5 to 92.4. In terms of individual domain scores, the three highest scoring domains were the Environment domain (*M* = 88.0), followed by the Moving In domain (*M* = 87.4), and the Care and Services domain (*M* = 86.6). The Facility Culture domain (*M* = 77.7) and the Caregivers domain (*M* = 73.0) received mid-level scores, aligning closely with the overall score. The two lowest-scoring domains were the Spending Time domain (*M* = 68.3) and the Meals and Dining domain (*M* = 66.8).

**Table 1:** Summary statistics of resident satisfaction (*N *= 752).

Variables	Mean (*SD*)	Min	Max
**Overall**	76.5 (5.9)	54.5	92.4
**Environment**	88.0 (5.8)	67.1	100.0
**Moving in**	87.4 (8.0)	56.6	100.0
**Care and services**	86.6 (6.2)	62.3	100.0
**Facility culture**	77.7 (8.2)	51.6	97.1
**Caregivers**	73.0 (6.0)	52.9	89.3
**Spending time**	68.3 (6.7)	45.8	88.0
**Meals and dining**	66.8 (10.3)	33.2	100.0

*Note*. *SD* = standard deviation.

Table 2 shows the summary statistics of NHA/DON turnover and control variables. Between 2015 and 2017, almost two-thirds of NHs (64.3%) reported any NHA turnover. Particularly, 27.3% had one NHA turnover, and 37.0% had two or more NHA turnovers. Similarly, 68.1% of NHs reported any DON turnover. Approximately one-third of NHs (31.9%) had one DON turnover, and 36.2% had two or more DON turnovers.

**Table 2:** Summary statistics of explanatory variables (*N *= 752).

Variables	Mean (*SD*) or percentage
**Key predictors**	
One NHA turnover	27.3
Two or more NHA turnovers	37.0
One DON turnover	31.9
Two or more DON turnovers	36.2
**Structural characteristics**	
Number of certified beds	93.5 (42.0)
% private rooms	44.1
For-profit	78.2
Chain affiliation	67.3
CCRC affiliation	17.4
Presence of a memory care unit	20.0
Presence of other special care units	6.4
Urban location	69.8
**Financial resource characteristics**	
Occupancy rate	83.0 (12.1)
Medicare payer mix	11.3
Medicaid payer mix	59.1
**Staff characteristics**	
RN HPRD	0.6 (0.4)
LPN HPRD	0.9 (0.3)
CNA HPRD	2.2 (0.5)
Social worker HPRD	0.1 (0.1)
Activity staff HPRD	0.2 (0.1)
Food service staff HPRD	0.7 (0.5)
Use of agency nursing staff	21.4
Licensed nurse retention rate	68.3
CNA retention rate	61.0
**Resident characteristics**	
Average age	78.4 (6.4)
Female	66.9
BIPOC	14.0
Acuity index	10.3 (1.1)
Residents with depression	52.6
Residents with dementia	45.3
Residents with psychiatric illness	41.1
Residents with intellectual disability	1.8

*Note*. BIPOC = Black, Indigenous, and People of Color; CAN = certified nursing assistants; CCRC = continuing care retirement community; DON = director of nursing; HPRD = hours per resident days; LPN = licensed practical nurses; NHA = nursing home administrator; RN = registered nurses; *SD* = standard deviation. Statistics on the analytic sample include adjustments for missing data. Acuity index is a continuous score indicating residents’ physical ability to perform activities of daily living and the need for special treatment.

In terms of other NH characteristics, NHs in the analytic sample have 94 licensed beds on average. The majority of NHs were for-profit (78.2%), chain-affiliated (67.3%), and located in an urban area (69.8%). The annual occupancy rate was 83.0%, and the majority of residents rely on Medicaid (59.1%). Residents receive the most hours of care from CNAs (2.2 hr) on a given day. More than one-fifth (21.4%) of NHs use agency nursing staff. The annual retention rate for licensed nurses is 68.3% and 61.0% for CNAs. Looking at the profile of NH residents across Ohio, the average age was 78 years. Residents were predominantly female (66.9%), and more than half of NH residents (52.6%) reported having depression.

### Main analyses: multiple linear regression

Table 3 shows the results of eight regression models that include four turnover predictors, while controlling for other variables. The coefficient, standard error, and significance level are reported. The last row for each regression model indicates adjusted *R*-squared values, ranging from 0.08 to 0.36.

**Table 3:** Results of linear regression for resident satisfaction scores (*N *= 752).

	**Resident satisfaction scores**							
	Overall	Environment	Moving in	Care and services	Facility culture	Caregivers	Spending time	Meals and dining
	*β* (*SE*)	*β* (*SE*)	*β* (*SE*)	*β* (*SE*)	*β* (*SE*)	*β* (*SE*)	*β* (*SE*)	*β* (*SE*)
One NHA turnover	0.39 (0.47)	1.00* (0.46)	−0.02 (0.71)	−0.03 (0.58)	0.64 (0.68)	0.56 (0.53)	0.12 (0.59)	−0.43 (0.87)
Two or more NHA turnovers	0.33 (0.49)	0.62 (0.48)	0.78 (0.74)	1.24* (0.61)	0.01 (0.71)	0.74 (0.55)	0.16 (0.62)	−0.93 (0.91)
One DON turnover	−0.47 (0.46)	−0.54 (0.44)	0.56 (0.69)	−0.04 (0.57)	−0.25 (0.66)	−1.01 (0.52)	−0.24 (0.58)	−0.07 (0.85)
Two or more DON turnovers	−1.14* (0.49)	−1.01* (0.48)	−0.15 (0.74)	−0.45 (0.61)	−1.06 (0.71)	−1.80** (0.56)	−0.95 (0.62)	−1.13 (0.91)
Controls	Y	Y	Y	Y	Y	Y	Y	Y
Adjusted *R*^2^	0.33	0.36	0.19	0.08	0.28	0.20	0.19	0.25

*Note*. DON = director of nursing; NHA = nursing home administrator; *SE* = standard error. All regression models are controlled for number of beds, percentage of private rooms, ownership status, chain affiliation, continuing care retirement community affiliation, presence of a dementia special care unit/other special care units, geographical location, occupancy rate, Medicare and Medicaid payer-mix, hours per resident day for registered nurses/licensed practical nurses/certified nursing assistants/food service staff/activity staff/social worker, use of agency nursing staff, retention rate of licensed nurse/certified nursing assistants, residents’ average age, gender, race/ethnicity, acuity index, percentage of residents’ with depression/dementia/psychiatric illness/intellectual disability. Acuity index is a continuous score indicating residents’ physical ability to perform activities of daily living and the need for special treatment. Adjusted *R*^2^ represents the explanatory power of the adjusted model, which includes both the key predictors and all control variables.

* *p* < .05.

** *p* < .01.

None of the NHA turnover measures are statistically associated with overall resident satisfaction, while they are positively associated with two individual domain scores. For example, NHs with one NHA turnover have a higher score in the Environment domain by 1.00% points (*p* < .05). NHs with two or more NHA turnovers have a higher score in the Care and Services domain by 1.24% points (*p* < .05).

One DON turnover is not statistically associated with either overall or individual resident satisfaction domains. However, two or more DON turnovers are associated with a lower overall resident satisfaction score by 1.14% points (*p* < .05), as well as lower scores of two individual domains. Specifically, NHs with two or more DON turnovers have a lower score in the Environment domain by 1.01% points (*p* < .05) and a lower score in the Caregivers domain by 1.80% points (*p* < .01). Results for the control variables are presented in Supplementary Table 3.

## Discussion and implications

This study examined the associations between the NHA/DON turnover and NH resident satisfaction scores. NHA turnover was not associated with the overall resident satisfaction score, and only two individual domain scores showed statistically significant associations. Yet, both of these suggest that higher NHA turnover is associated with higher satisfaction. In contrast, DON turnover, especially NHs with two or more DONs in the past two years, was associated with lower overall satisfaction and two of the seven individual domain scores. As hypothesized, one of these domains was related to caregivers. In conclusion, the findings partially supported H1 and H3 and fully supported H2.

The findings are consistent with several previous studies on NH consumer experiences. For example, Kennedy (2023) found no significant association between NHA turnover and resident satisfaction scores; however, NHs without DON turnover had marginally but significantly higher overall resident satisfaction scores compared to those with DON turnover. Similarly, an increasing number of DON changes is associated with lower overall resident satisfaction, while no significant association is observed between the number of NHA changes and overall satisfaction (Kwon et al., 2025). Shippee et al. (2015) found that having NHA turnover is not associated with overall quality of life score but was significantly related to worse Mood domains among Minnesota NH residents.

This study holds more nuanced insights into leadership turnover. Interestingly, having one DON turnover is not significantly associated with overall satisfaction or the seven individual satisfaction domains. This finding aligns with a prior study using the same Ohio resident satisfaction dataset, which reported that NHs in the third quartile of CNA retention had higher overall resident satisfaction scores and seven individual domain scores compared to those in the highest quartile (Kennedy, 2023). This suggests that beyond a certain threshold, maintaining high workforce stability does not necessarily translate into better resident experiences. This implies that the relationship between workforce stability and resident satisfaction is complex and may follow a non-linear pattern.

The findings that frequent DON turnover is associated with lower overall resident satisfaction and lower scores on the Environment and Caregivers domains can be interpreted through the lens of the Person-Environment (P-E) Fit theory. When DON changes are frequent, the consistency of nursing oversight could lead to disrupted coordination of day-to-day care (Kwon et al., 2025), thereby increasing environmental pressure beyond what residents with diminished competence can comfortably manage. This misalignment between residents’ functional limitations and an unstable care environment may manifest as reduced satisfaction, particularly in domains that reflect direct environment and staff interactions with residents.

Surprisingly, NHA turnover is positively associated with two individual domains. Specifically, one NHA turnover is associated with a higher Environment score, and two or more NHA turnovers are associated with a higher Care and Services score. Although NHA turnover was expected to negatively affect resident satisfaction across domains, these positive associations may indicate that leadership change sometimes brings renewed attention to tangible aspects of care delivery and the physical environment, resulting in short-term improvements in these areas. Alternatively, the cross-sectional nature of the current study may have captured a “fresh start” effect, in which new leadership temporarily improves perceptions of certain domains. Future longitudinal research is needed to determine whether these positive associations reflect temporary adjustments following leadership change or sustained improvements in care quality.

The findings that DON turnover negatively affects resident satisfaction carry important practice and policy implications. At the practice level, NHs should prioritize strategies to retain DONs, such as providing competitive salary, reducing administrative burdens, and fostering empowering work environments, while implementing robust succession planning to minimize disruptions during leadership transitions (Duffield et al., 2011; Kash et al., 2010; Warden et al., 2021). From a policy perspective, regulatory agencies could incorporate DON turnover rates into quality monitoring systems and incentivize NHs that demonstrate stable nursing leadership through funding or recognition programs.

### Limitations

As with all research, this study has several limitations. First, this study is limited to Ohio. While Ohio has a high number of NHs and reliable datasets on long-term care (Applebaum et al., 2020), the findings are not generalizable to other states. Long-term care systems vary considerably across states due to differences in policies, funding structures, workforce availability, and resident demographics (Bowblis et al., 2023; Brazier et al., 2023; Peterson et al., 2021). As a result, the applicability of these findings beyond Ohio is limited. Future research could examine whether similar findings emerge in other states.

Second, information on residents’ length of stay and cognitive status was not available to control. The length of stay could influence resident satisfaction, as it reflects residents’ care expectations, needs, goals, and acuity levels (Shippee et al., 2015, 2023). Short-stay residents tend to be more mobile, less medically complex, and have defined discharge goals (Atkins et al., 2017), which may lead to higher initial satisfaction due to greater independence and optimism about recovery. In contrast, long-stay residents typically have severe chronic conditions, cognitive impairments, or disabilities (Atkins et al., 2017), and their differing expectations and care needs may result in lower satisfaction ratings. In addition, given the high prevalence of dementia among the NH population and its substantial influence on residents’ quality of life and care experiences (Shippee et al., 2023), the absence of this information may limit the accuracy and generalizability of the findings. Future research should incorporate these variables and consider stratified analyses to better capture heterogeneity in residents’ experiences.

Lastly, this study does not account for baseline satisfaction score or expectations about NH care when assessing residents’ satisfaction levels. Resident satisfaction is heavily influenced by individuals’ preexisting expectations regarding the services they anticipate receiving (Parasuraman et al., 1988). For example, even when two residents receive identical care, a person with high initial expectations may report lower satisfaction scores compared to someone with more modest expectations. Measuring residents’ expectations before their admission to a NH and then comparing these with their actual perception of care quality after living in the NH could provide deeper insights into how initial expectations shape their actual experiences with care services.

## Conclusions

In conclusion, this study highlights that DON may have a greater impact on residents’ care experiences than NHA turnover. Specifically, while limited DON turnover (e.g., a single change) was not associated with significantly lower resident satisfaction, frequent DON turnover (two or more changes) was associated with lower overall resident satisfaction as well as two individual domains. As the primary supervisor of nursing care delivery, the DON plays a central yet often underrecognized role in shaping residents’ day-to-day experiences. These findings underscore the importance of stable DON leadership in fostering positive consumer experience in NHs.

## Supplementary Material

gnag106_Supplementary_Data

## Data Availability

Data or analytic methods are available upon request. The study reported was not pre-registered.
