# Supplementary material for: Associations between nursing home leadership turnover and resident satisfaction
Source: Gerontologist. 2026 May 11;66(7):gnag106. doi: 10.1093/geront/gnag106 (PMC13284986; doi:10.1093/geront/gnag106)
Supplement: gnag106_Supplementary_Data [file gnag106_supplementary_data.pdf]

Title: Associations Between Nursing Home Leadership Turnover and Resident Satisfaction

Author names and affiliations: Jenny H. Kwon, PhD,[1,2] & John R. Bowblis, PhD,[3,4]

[1]Philip R. Lee Institute for Health Policy Studies, University of California, San Francisco, CA, USA
[2]Department of Epidemiology & Biostatistics, University of California, San Francisco, CA, USA
[3]Department of Economics, Farmer School of Business, Miami University, Oxford, OH, USA
[4]Scripps Gerontology Center, Miami University, Oxford, OH, USA

Corresponding author: Jenny H. Kwon, PhD
Philip R. Lee Institute for Health Policy Studies
Department of Epidemiology & Biostatistics
University of California, San Francisco, CA 94158, USA
E-mail: Jenny.Kwon@ucsf.edu
Phone: +1 812-272-5272
ORCID: 0000-0002-7167-9562

Co-author:
John R. Bowblis, PhD
Department of Economics
Farmer School of Business
Miami University, Oxford, OH 45056, USA
E-mail: bowblijr@miamioh.edu
ORCID: 0000-0003-4776-4182

Running title: Nursing Home Leadership Turnover and Resident Satisfaction

Supplementary Material

Supplementary Table 1. 2017 Ohio Nursing Home Resident Satisfaction Survey Instrument

Domain	Items
**Environment**	1. Is it very clean here?<br>2. Is it easy for you to get around in your room?<br>3. Can you enjoy the outdoors when you want to?<br>4. Do you feel you have enough privacy?<br>5. Can you find a place to be alone when you want to be alone?<br>6. Are your personal items safe here?<br>7. Do you feel safe here?
**Moving In**	8. Do you remember what it was like when you first moved in here?<br>9. Were you given enough help to learn how things work here?<br>10. Did you feel warmly welcomed as a new resident?
**Care and Services**	11. Are your preferences about daily routines carried out?<br>12. Do the people who work here give you enough time to do the things you can do for yourself?<br>13. Have you gotten or are you getting special therapies, like physical therapy, occupational therapy or speech therapy, while living at this nursing home?<br>14. Did the therapists help you set goals?<br>15. Did the therapy help you meet your goals?<br>16. Did (do) you know who to speak to about your therapy progress?
**Facility Culture**	17. Are you encouraged to speak up about things you don't like here?<br>18. Are your concerns taken care of in a timely way?<br>19. Are you involved in decisions about your care?<br>20. Do the people who work here seem happy to work here?<br>21. Do the people who work here go above and beyond to give you a good life?<br>22. Do you feel included in life here?<br>23. Are you friends with anyone who lives here?<br>24. Would you highly recommend this nursing home to a family member or friend?
**Caregivers**	25. Are the people who work here knowledgeable about your medical conditions and treatments?<br>26. Do the people who work here do things the way you want them done?<br>27. Do the people who work here check on your often enough to see if you need anything?<br>28. Are the people who work here gentle with your care?<br>29. Do the people who work here come quickly anytime you call or ask for help?<br>30. Do the people who work here ever get angry at you?<br>31. Do the people who work here tell you what they are doing when they care for you?<br>32. Do the same people take care of you most of the time?

**Spending Time**	33. Do you usually enjoy how you spend your time?<br>34. Do you have something to look forward to most days?<br>35. Do the people who work here keep you connected to the community?<br>36. Are you given plenty of opportunities to do things that are meaningful to you?<br>37. Do you like the activities that are provided here?<br>38. Do you spend too much time waiting for things?<br>39. Does this facility [or facility name] provide enjoyable things to do on the weekends?
**Meals and Dining**	40. Do you get your favorite foods here?<br>41. Does the menu change often enough?<br>42. Do you have input into the food that is served?<br>43. Do you like the food here?<br>44. Do you look forward to mealtimes?

Response options: Generally, yes (1); Generally, no (2); Don’t know/Not applicable (3).

Source: Vital Research. (2018b). 2017 Ohio long-term care resident satisfaction survey: Survey narrative report. *Vital Research,* 1–46.

Supplementary Table 2. Summary of Measures

Name of Variables	Level of Measurement in the Regression Models	Source
**Outcome Variables**		
Resident satisfaction scores	Continuous: 0 (Low)-100 (High)	The 2017 Ohio Nursing Home Resident Satisfaction Survey
Overall		
Environment		
Moving In		
Care and Services		
Facility Culture		
Caregivers		
Spending Time		
Meals and Dining		
**Key Predictors**		
NHA turnover	One turnover, Two or more (2+) turnover between 2015 and 2017; (Ref=No NHA turnover)	The 2017 Ohio Biennial Survey of Long-Term Care Facilities
DON turnover	One change, Two or more (2+) turnover between 2015 and 2017; (Ref=No DON turnover)	
**Control Variables**		
**Structural Characteristics**		
Number of certified beds	Continuous	Medicaid Cost Report (MCR)
Private rooms	(Number of private rooms/Total rooms on December 31, 2017)*100;<br>Continuous: 0 (Low)-100 (High)	The 2017 Ohio Biennial Survey of Long-Term Care Facilities
Ownership status	For-profit<br>(Ref=Not-for-profit + Government-owned)	Certification and Survey Provider Enhanced Reports (CASPER)
Chain affiliation	Yes (Ref=No)	
CCRC affiliation		
Presence of a memory care unit		
Presence of other special care units		
Geographic location	Urban (Ref=Not urban)	
**Financial Resource Characteristics**		
Occupancy rate	Total resident days/Total bed days*100;<br>Continuous: 0 (Low)-100 (High)	

Medicare payer mix	Medicare days/Total resident days*100; Continuous: 0 (Low)-100 (High)	Medicaid Cost Report (MCR)
Medicaid payer mix	(Total Medicaid days + Managed care days)/Total resident days*100; Continuous: 0 (Low)-100 (High)	
**Staff Characteristics**		
RN HPRD	Total RN hours paid/Total resident days in 2017; Continuous	Medicaid Cost Report (MCR)
LPN HPRD	Total LPN hours paid/Total resident days in 2017; Continuous	
CNA HPRD	Total CNA hours paid/Total resident days in 2017; Continuous	
Food service staff HPRD	Total food service staff hours paid/Total resident days in 2017; Continuous	
Activity staff HPRD	Total activity staff hours paid/Total resident days in 2017; Continuous	
Social worker HPRD	Total social worker hours paid/Total resident days in 2017; Continuous	
Use of agency nursing staff (RN, LPN, and CNAs)	Percentage of NHs using agency nursing staff in 2017; Continuous	
Licensed nurses (RN+LPN) retention rate	(Number of staff who remain employed at the end of 2017/Total number of staff employed at the start of 2017)*100; Continuous: 0 (Low)-100 (High)	The 2017 Ohio Biennial Survey of Long-Term Care Facilities
CNA retention rate	(Number of staff who remain employed at the end of 2017/Total number of staff employed at the start of 2017)*100; Continuous: 0 (Low)-100 (High)	
**Resident Characteristics**		
Average age	The average age of residents; Continuous	LTCFocus
Female	The percentage of female residents; Continuous	
BIPOC	The percentage of BIPOC residents; Continuous	
Acuity index	Pre-calculated index score that consists of two measures: 1) a score of residents' physical ability to perform activities of daily living and 2) a score reflecting the special treatment and clinical needs; Continuous; A higher score indicates a greater need for assistance with activities of daily living and specialized care services	Certification and Survey Provider Enhanced Reports (CASPER)
Residents with depression	The percentage of residents with specific symptoms; Continuous	
Residents with dementia		
Residents with psychiatric illness		
Residents with intellectual disability		

Note: NHA=Nursing home administrator; DON=Director of nursing; CCRC=Continuing care retirement community; RN=Registered nurse; LPN=Licensed practical nurse; CNA=Certified nursing assistant; HPRD=Hours per resident day; BIPOC=Black, Indigenous, or People of Color. Acuity index is a continuous score indicating residents' physical ability to perform activities of daily living and the need for special treatment.

Supplementary Table 3. Results of Regression for Control Variables (N=752)

	Resident Satisfaction Scores							
	Overall	Environment	Moving In	Care and Services	Facility Culture	Caregivers	Spending Time	Meals and Dining
	β (SE)	β (SE)	β (SE)	β (SE)	β (SE)	β (SE)	β (SE)	β (SE)
Number of certified beds	-0.02** (0.00)	-0.01* (0.00)	-0.02* (0.01)	-0.00 (0.01)	-0.03*** (0.01)	-0.01* (0.01)	-0.01 (0.01)	-0.03** (0.01)
Private rooms (%)	0.02** (0.01)	0.05*** (0.01)	0.01 (0.01)	0.01 (0.01)	0.02* (0.01)	0.01 (0.01)	0.01 (0.01)	0.00 (0.01)
For-profit ownership (Ref = Not-for-profit + Government-owned)	-0.58 (0.57)	-0.72 (0.55)	0.85 (0.85)	-0.30 (0.71)	-0.88 (0.82)	0.39 (0.65)	-0.56 (0.72)	-1.89[c] (1.05)
Chain affiliation	-0.13 (0.40)	-0.14 (0.39)	-1.19* (0.60)	0.12 (0.50)	0.01 (0.57)	0.08 (0.45)	-0.33 (0.51)	-0.95 (0.74)
CCRC affiliation	-1.42* (0.57)	-0.88[c] (0.53)	-2.10* (0.82)	-0.92 (0.70)	-1.41[c] (0.78)	-1.15 (0.64)	-1.24[c] (0.71)	-1.71[c] (1.03)
Presence of a memory care unit	-0.15 (0.49)	-0.08 (0.47)	-0.68 (0.73)	1.35* (0.60)	0.17 (0.70)	-0.30[c] (0.55)	-0.27 (0.61)	-0.82 (0.90)
Presence of other special care units	0.01 (0.76)	0.22 (0.73)	0.30 (1.13)	-0.82 (0.94)	-0.84 (1.08)	-0.05 (0.85)	-0.50 (0.95)	1.30 (1.39)
Urban location (Ref=Not urban)	-2.04*** (0.44)	-1.83*** (0.42)	-1.79** (0.65)	-1.19* (0.54)	-2.96*** (0.63)	-1.69*** (0.49)	-1.79** (0.55)	-2.59** (0.81)
Occupancy rate	-0.01 (0.02)	-0.05** (0.02)	0.02 (0.02)	-0.02 (0.02)	-0.03 (0.02)	-0.01 (0.02)	0.05* (0.02)	0.04 (0.03)
Medicare payer mix (%)	0.00 (0.02)	0.01 (0.02)	-0.05 (0.04)	0.03 (0.03)	-0.04 (0.03)	-0.00 (0.03)	-0.03 (0.03)	0.10* (0.04)

	Resident Satisfaction Scores							
	Overall	Environment	Moving In	Care and Services	Facility Culture	Caregivers	Spending Time	Meals and Dining
	β (SE)	β (SE)	β (SE)	β (SE)	β (SE)	β (SE)	β (SE)	β (SE)
Medicaid payer mix (%)	-0.02<br>(0.01)	-0.03*<br>(0.01)	-0.01<br>(0.02)	-0.01<br>(0.02)	-0.02<br>(0.02)	-0.01<br>(0.01)	-0.03^{c}<br>(0.02)	-0.01<br>(0.02)
RN HPRD	0.46<br>(0.67)	-0.44<br>(0.65)	0.79<br>(1.01)	0.88<br>(0.83)	0.43<br>(0.97)	0.94<br>(0.76)	-0.62<br>(0.85)	0.71<br>(1.24)
LPN HPRD	0.04<br>(0.76)	-0.21<br>(0.73)	-0.24<br>(1.13)	0.27<br>(0.94)	0.22<br>(1.08)	-0.41<br>(0.86)	-0.93<br>(0.96)	1.63<br>(1.39)
CNA HPRD	0.22<br>(0.46)	-0.57<br>(0.45)	-0.16<br>(0.69)	0.31<br>(0.57)	0.13<br>(0.66)	1.07<br>(0.52)	0.07<br>(0.58)	1.10<br>(0.85)
Social worker HPRD	4.45<br>(3.26)	2.47<br>(3.08)	15.80***<br>(4.75)	1.23<br>(4.05)	6.96<br>(4.56)	2.48*<br>(3.68)	9.63*<br>(4.11)	-1.32<br>(5.91)
Activities staff HPRD	1.52<br>(1.65)	N/A	N/A	-0.49<br>(1.89)	N/A	1.16<br>(1.87)	7.91<br>(1.92)	N/A
Food service staff HPRD	0.62<br>(0.41)	N/A	N/A	N/A	N/A	0.17<br>(0.46)	N/A	1.44*<br>(0.69)
Use of agency nursing staff (%)	-1.09**<br>(0.39)	-0.53<br>(0.38)	-0.85<br>(0.58)	-0.91^{c}<br>(0.48)	-1.70**<br>(0.56)	-1.23<br>(0.44)	-0.34***<br>(0.49)	-2.03**<br>(0.72)
Nursing staff retention rate	0.01<br>(0.01)	0.01<br>(0.01)	0.01<br>(0.01)	-0.00<br>(0.01)	0.01<br>(0.01)	0.01**<br>(0.01)	0.00<br>(0.01)	0.02<br>(0.02)
CNA retention rate	-0.00<br>(0.01)	0.00<br>(0.01)	-0.01<br>(0.02)	-0.00<br>(0.01)	-0.01<br>(0.02)	0.00<br>(0.01)	0.00<br>(0.01)	-0.01<br>(0.02)
Average age	0.23***<br>(0.06)	0.28***<br>(0.06)	0.19*<br>(0.09)	0.10<br>(0.07)	0.32**<br>(0.08)	0.18**<br>(0.07)	0.16*<br>(0.07)	0.23*<br>(0.11)

	Resident Satisfaction Scores							
	Overall	Environment	Moving In	Care and Services	Facility Culture	Caregivers	Spending Time	Meals and Dining
	β (SE)	β (SE)	β (SE)	β (SE)	β (SE)	β (SE)	β (SE)	β (SE)
Female (%)	0.03<br>(0.02)	0.00<br>(0.02)	0.05<br>(0.04)	0.00<br>(0.03)	0.05<br>(0.03)	0.01<br>(0.03)	0.05[c]<br>(0.03)	0.01<br>(0.04)
BIPOC (%)	-0.04**<br>(0.01)	-0.01<br>(0.01)	-0.07***<br>(0.02)	-0.03*<br>(0.02)	-0.06**<br>(0.02)	-0.04**<br>(0.01)	-0.01<br>(0.02)	-0.10***<br>(0.02)
Acuity index	0.03<br>(0.17)	0.40*<br>(0.17)	0.05<br>(0.26)	-0.21<br>(0.21)	0.10<br>(0.25)	-0.15<br>(0.20)	0.33<br>(0.22)	-0.35<br>(0.32)
Residents with depression (%)	-0.00<br>(0.01)	0.01<br>(0.01)	-0.01<br>(0.01)	-0.00<br>(0.01)	-0.00<br>(0.01)	-0.01<br>(0.01)	-0.01<br>(0.01)	-0.00<br>(0.02)
Residents with dementia (%)	0.01<br>(0.02)	0.01<br>(0.01)	-0.01<br>(0.02)	0.02<br>(0.02)	-0.02<br>(0.02)	0.04*<br>(0.02)	-0.00<br>(0.02)	0.05[c]<br>(0.03)
Residents with psychiatric illness (%)	-0.01<br>(0.01)	-0.02[c]<br>(0.01)	-0.05**<br>(0.02)	-0.01<br>(0.02)	-0.00<br>(0.02)	-0.01<br>(0.01)	-0.02<br>(0.02)	0.02<br>(0.02)
Residents with intellectual disability (%)	-0.07<br>(0.07)	-0.06<br>(0.07)	-0.21*<br>(0.10)	-0.10<br>(0.09)	-0.14<br>(0.10)	0.05<br>(0.08)	-0.08<br>(0.09)	-0.13<br>(0.13)

Note: SE=Standard error; CCRC=Continuing care retirement community; RN=Registered nurse; HPRD=Hours per resident day; LPN=Licensed practical nurse; CNA=Certified nursing assistant; BIPOC=Black, Indigenous, and People of Color; The acuity index is a continuous measure, with higher scores indicating a greater need for assistance with activities of daily living and specialized care services; [c]p<0.1, *p<.05, **p<.01, ***p<.001.
